# Influence of Speed, Ground Surface and Shoeing Condition on Hoof Breakover Duration in Galloping Thoroughbred Racehorses

**DOI:** 10.3390/ani11092588

**Published:** 2021-09-03

**Authors:** Kate Horan, James Coburn, Kieran Kourdache, Peter Day, Dan Harborne, Liam Brinkley, Henry Carnall, Lucy Hammond, Mick Peterson, Sean Millard, Thilo Pfau

**Affiliations:** 1 Department of Clinical Science and Services, The Royal Veterinary College, Hawkshead Lane, Brookmans Park, Hertfordshire AL9 7TA, UK; pday@rvc.ac.uk (P.D.); smillard3@rvc.ac.uk (S.M.); tpfau@rvc.ac.uk (T.P.); 2 James Coburn AWCF Ltd., 14 Church Lane Close, Barton Mills, Bury St Edmunds IP38 6AX, UK; jamescoburndhs@hotmail.co.uk (J.C.); daneastern13@gmail.com (D.H.); liamdbrinkley@hotmail.co.uk (L.B.); henrycarnall@hotmail.co.uk (H.C.); 3 The British Racing School, Snailwell Road, Newmarket CB8 7NU, UK; KieranK24@hotmail.com (K.K.); lucyhammond99@gmail.com (L.H.); 4 Biosystems and Agricultural Engineering, University of Kentucky, Lexington, KY 40506-0503, USA; mick.peterson@uky.edu

**Keywords:** racehorse, hoof, breakover, gallop, shoeing condition, surface, speed

## Abstract

**Simple Summary:**

In the stride cycle of a horse, there is a period of time when the hoof pushes off from the ground surface and rotates through an angle of approximately 90 degrees before it is lifted off. This time period is known as hoof breakover. Using slow-motion video footage, this study measured breakover duration in retired Thoroughbred racehorses galloping at a range of speeds on two surfaces (artificial and turf) in four shoeing conditions (aluminium, barefoot, GluShu and steel). Hooves from different limbs were assessed separately in this asymmetric gait. Increasing speed was correlated with decreasing breakover duration, and this trend was more enhanced in the hindlimbs than in the forelimbs at high gallop speeds. Breakover duration was faster on the artificial surface compared to the turf surface for all limbs, under the ground conditions studied. The first limb to contact the ground surface after the suspension phase (the ‘non-leading’ hindlimb), was additionally influenced by shoeing condition and an interaction that occurred between shoeing condition and speed. Determining parameters that alter breakover duration will be important for lowering the risk of musculo-skeletal injuries, optimising gait quality and improving performance in galloping racehorses during both training and racing.

**Abstract:**

Understanding the effect of horseshoe–surface combinations on hoof kinematics at gallop is relevant for optimising performance and minimising injury in racehorse–jockey dyads. This intervention study assessed hoof breakover duration in Thoroughbred ex-racehorses from the British Racing School galloping on turf and artificial tracks in four shoeing conditions: aluminium, barefoot, aluminium–rubber composite (GluShu) and steel. Shoe–surface combinations were tested in a randomized order and horse–jockey pairings (*n* = 14) remained constant. High-speed video cameras (Sony DSC-RX100M5) filmed the hoof-ground interactions at 1000 frames per second. The time taken for a hoof marker wand fixed to the lateral hoof wall to rotate through an angle of 90 degrees during 384 breakover events was quantified using Tracker software. Data were collected for leading and non-leading forelimbs and hindlimbs, at gallop speeds ranging from 23–56 km h^−1^. Linear mixed-models assessed whether speed, surface, shoeing condition and any interaction between these parameters (fixed factors) significantly affected breakover duration. Day and horse–jockey pair were included as random factors and speed was included as a covariate. The significance threshold was set at *p* < 0.05. For all limbs, breakover times decreased as gallop speed increased (*p* < 0.0005), although a greater relative reduction in breakover duration for hindlimbs was apparent beyond approximately 45 km h^−1^. Breakover duration was longer on turf compared to the artificial surface (*p* ≤ 0.04). In the non-leading hindlimb only, breakover duration was affected by shoeing condition (*p* = 0.025) and an interaction between shoeing condition and speed (*p* = 0.023). This work demonstrates that speed, ground surface and shoeing condition are important factors influencing the galloping gait of the Thoroughbred racehorse.

## 1. Introduction

The stride of a horse is punctuated by intervals when limbs transition out of stance and into propulsive phases and the heels of the hooves lift off and pivot about the toes. These periods in time may be referred to as hoof breakover. Depending on gait, breakover may occur concurrently amongst pairs of hooves (trot, pace), or at different stages of a stride (walk, canter, gallop, tölt), with a duration that may reflect whether the gait is symmetric (walk, trot, pace, tölt) or asymmetric (canter, gallop) [1,2]. Although gait is expected to impose a fundamental constraint on the rate of breakover, through its influence on speed, farriery manipulations of the hoof and environmental surface conditions are also relevant considerations.

Medio-lateral and dorso-palmar/plantar balance of a horse’s hoof may be adjusted by trimming and shoeing to influence hoof impact, loading and, hence, the preparation of the distal limb for push-off during breakover [3]. In addition, farriery interventions aimed at altering breakover, specifically, are relevant for reducing stress and strain on joints or tendons, and may additionally improve joint alignment and gait, while also limiting overload injuries [4]. This is pertinent for orthopaedic cases, such as horses with laminitis, injuries to flexor tendons or those with navicular lesions that require support for palmar structures. In performance horses, discipline specific alterations to breakover may also be important. At the beginning of breakover, the deep digital flexor tendon exerts peak forces on the navicular bone [5] and flexes the interphalangeal joints. However, towards the end of breakover when only the toe contacts the ground, force is transferred to the ground to promote limb propulsion. In this latter stage of breakover, load is also transferred to the dorsal hoof wall and the tip of the distal phalanx [6,7]. Elastic recoil during push-off is aided by the release of energy that had been stored in the superficial flexor tendon and suspensory ligament [8]; during gallop, in vivo strains of 16% in the superficial digital flexor tendon have been reported [9]. In galloping horses, breakover is thought to take place in the time period approximately 85 to 100% through stance [10].

Increased breakover durations have been linked to increased hoof toe lengths [11,12,13], as a longer toe is associated with a longer moment arm at the distal interphalangeal joint. Horses with low heels and long toes often lack phalangeal alignment, and it is plausible that these horses might benefit from trimming to shorten the toe or shoes with the toe section positioned more caudally. Placing the toe region of a horseshoe more caudally causes the moment arm of the ground reaction force on the distal interphalangeal joint to reduce, and should therefore accelerate breakover. However, the peak moment and force on the navicular bone may not be greatly influenced because breakover starts earlier and at a high ground reaction force [14,15]. Nevertheless, breakover duration was not found to be significantly different when rocker-toe, rolled toe, or square toe shoes were compared to conventional plain steel shoes in sound horses trotting on firm surfaces [16,17], but it is possible that results would differ in horses with pathologies. Shoulder problems, carpal fractures and navicular disease typically increase breakover times, possibly due to restricted mechanical movement or pain associated with limb orientation in the latter part of stance [11]. In contrast, breakover duration may be reduced when wedges are applied to the heel region of hooves [18,19] and heel elevation can lessen the force on the navicular bone [5,11,20]. This reflects the movement of the point of force to the toe occurring later in stance and, along with a delayed unloading of the heel, breakover duration decreases [21]. It is worth noting, however, that most of these previous studies have focussed on trying to understand the influence of trimming and shoeing on breakover patterns at the walk and trot on firm surfaces. A better appreciation of how the dynamics of the hoof can be altered during breakover for horses in different disciplines, moving over different ground conditions in various shoeing conditions and at higher speeds, would be informative towards efforts to improve equine well-being and performance.

The present study sought to quantify breakover duration in galloping horses for the first time (to the authors’ knowledge) and to evaluate this in the context of different horseshoes and ground surfaces at a range of racing speeds. It also aimed to investigate whether differences between limbs (front versus hind, lead versus non-lead) could be identified. With increasing speed, we expected breakover duration to decrease, following on from previous observations of reduced stance times and duty factors in galloping horses with increasing speed [2,22,23]. We also hypothesised that ground surface would have the dominant effect on breakover duration due to the potential effect of surface properties on hoof landing angle and orientation during stance [24].

## 2. Materials and Methods

### 2.1. Ethics

Ethical approval for this study was received from the Royal Veterinary College Clinical Research Ethical Review Board (URN 2018 1841-2) and the participating jockeys, farriers and horse owners provided informed consent.

### 2.2. Horse and Jockey Participants

A convenience sample of 13 retired Thoroughbred racehorses at the British Racing School (BRS) in Newmarket, UK, were included in this study. The horses were in regular work, including gallop training, and were normally utilised for jockey education. They ranged in age from 6–20 years old, with heights from 15.3–16.3 hh, and they had masses between 421 and 504 kg. The horses were also included in a previously published study, where further details on individual horse body dimensions and hoof morphometrics are available [25]. All horses were considered sound by the jockeys, farriers and BRS senior management prior to data collection, and they are regularly checked by a veterinarian.

There were four participating jockeys. Jockey-1 is currently a racehorse trainer, but was previously a jockey, and has over 20 years of experience in the racing industry; Jockey-2 raced for 6 years, 10 years before the study, and currently works at the BRS as a riding instructor; Jockey-3 has been working in racing for approximately 3 years and currently works as a traveling head person, as well as riding 4 horses per day 6 days per week, ranging from yearlings to older horses; and Jockey-4 has a category A and point-to-point license, with in excess of 40 rides and 5–6 years of experience [25]. During trials, horse–jockey pairings were fixed, while shoe–surface conditions varied. One horse was ridden by two jockeys, giving rise to 14 possible horse–jockey pairings.

### 2.3. Trial Conditions

The horse–jockey dyads underwent randomized data collection trials on level artificial and turf surfaces in the following four shoeing conditions: (1) aluminium raceplates (Kerkhaert Aluminium Kings Super Sound horseshoes); (2) barefoot; (3) GluShus (aluminium–rubber composite horseshoes); and (4) steel shoes (Kerkhaert Steel Kings horseshoes). All horses had their hooves trimmed prior to data collection. After trimming, the toe axis and pastern were aligned. The three shoe types were fitted so the heels aligned with the widest part of the frog and the toe region of the shoes was aligned to the dorsal hoof wall. The rubber clips on the GluShus were removed, resulting in all three shoe types having a style with open heels and no clips. The shoes were secured with five copper coated mild steel nails. Typical shoe masses were 134 ± 26 g (mean ± 2 S.D., unless otherwise stated) for the aluminium shoes (*n* = 67), 191 ± 50 g for the GluShus (*n* = 56), and 333 ± 11 g for the steel shoes (*n* = 65). The artificial surface used was the Martin Collins Activ-Track, which comprises sand and CLOPF fibre. It is wax-coated, dust-free and designed for use in all weather conditions. It is frequently harrowed by the BRS during routine management but it does not require an irrigation system. Turf conditions during data collection ranged from ‘soft’ to ‘good-firm’. Full details of the weather on and preceding data collection days are available in [25]. In summary, in the 72 h preceding and inclusive of data collection days, mean temperature was 10.3 ± 10.3 °C, mean rainfall was 0.2 ± 0.4 mm and mean humidity was 80.9 ± 11.2% (*n* = 18).

The horses underwent a short period of adaptation to the shoeing conditions prior to data collection. This typically lasted around 10 min and involved the horse–jockey pairs walking and trotting over tarmac to the gallop tracks, before undertaking a warm-up over the surface they were about to be filmed galloping over. The warm-up consisted of walking, trotting, cantering and galloping in a straight line. Each horse took part up in up to four data collection trials per day (Tables S1 and S2), so they did not exercise for a duration beyond what is typical of a short riding session; this was approximately 15 min per trial, including the warm-up. A rest interval was provided between trials during which the shoeing condition was changed and/or filming equipment was moved between gallop tracks. In general, each trial consisted of two gallop runs in a given shoe–surface combination, to generate data for the horses galloping on left and right leads. However, some horses participated in additional gallop runs if they did not show the required lead, behaved in an undesired manner in a trial (such as bucking) or if equipment fell-off and needed to be reaffixed. Trials took place across multiple days for each horse–jockey dyad to acquire data for as many of the eight possible shoe–surface combinations as was feasible; limitations were imposed due to horse and jockey availability and routine turf accessibility restrictions implemented by the BRS to avoid ‘hard’ going.

### 2.4. Equipment and Filming Proceedure

**Figure 1:**
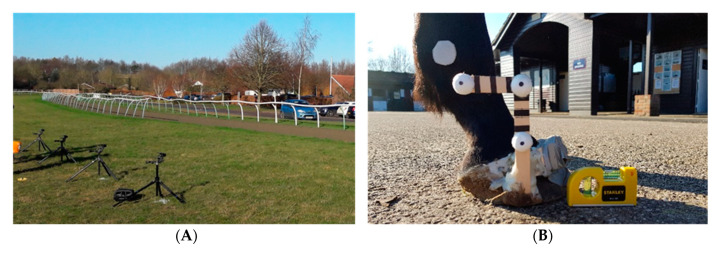
Filming set-up. (**A**) Four Sony DSC-RX100M5 cameras positioned 8.5 m from the centre of the track and spaced 3.5 m apart. (**B**) Hoof marker wand used as an aid for tracking hoof orientation through time in video footage.

The horses were filmed using four high-speed video cameras (Sony DSC-RX100M5) at 1000 frames per second, for an interval of approximately 3 s. The cameras were spaced 3.5 m apart, at a height of 75 cm; an arrangement that ensured the overall capture of at least one hoof strike per limb in each gallop run (Figure 1A). The total field of view was approximately 15 m.

This study required a visual cue from which to track hoof motion in the sagittal plane. Custom-made hoof marker wands were therefore created, similar to previous studies, e.g., [26,27], with a design that ensured they projected above the ground level even on soft surfaces. They consisted of two wooden sticks glued together at 90 degrees, supporting white polystyrene balls that could be easily detected when filming at a distance of approximately 8.5 m away from the horse and jockey. The hoof wands were secured to the lateral aspect of the right fore and right hind hooves of each horse using Superfast hoof adhesive (Figure 1B).

Jockeys were additionally provided with a GPS device (Holux RCV 3000) to carry in their pocket during trials. This device recorded their position every second, and from these data, speed during gallop runs could be quantified.

### 2.5. Data Processing

Video data were available for processing from 84 trials involving 13 horses (14 horse–jockey pairs) testing the eight possible shoe–surface combinations over 203 gallop runs. Occasionally, there were trials that did not generate any viable data due to the hoof marker wand breaking or becoming obscured by dirt kicked up by the horse, or because the horse ran close to the grass verge on the artificial track, where the wand was out of view. There were also two trials where breakover duration data were discounted because the horse was bucking or had become disunited. Breakover duration was quantified and included in analyses from a total of *n* = 384 hoof push-off events (*n* = 195 for forelimb data and *n* = 189 for hindlimb data) (Supplementary Material Tables S1 and S2).

Camera files were imported into Tracker Software, version 5.1.5 [28]. Using the Tracker protractor tool, an angle of 90 degrees was manually drawn on to each video image. The lower angle arm was orientated so it joined the upper two polystyrene markers on the hoof wand (Figure 1B) in the last camera frame before the start of breakover. This timing was immediately prior to any heel lift, and defined by the marker wand having no movement in the preceding frame and beginning rotation in the cranial direction in the following frame. Each video was subsequently played until the marker wand completed a rotation angle of 90 degrees, into an orientation parallel to the upper angle arm. The number of frames taken to reach this position was noted and used to calculate breakover duration (in frames). For each gallop run, the breakover duration of one forelimb and one hindlimb hoof strike was quantified.

**Figure 2:**
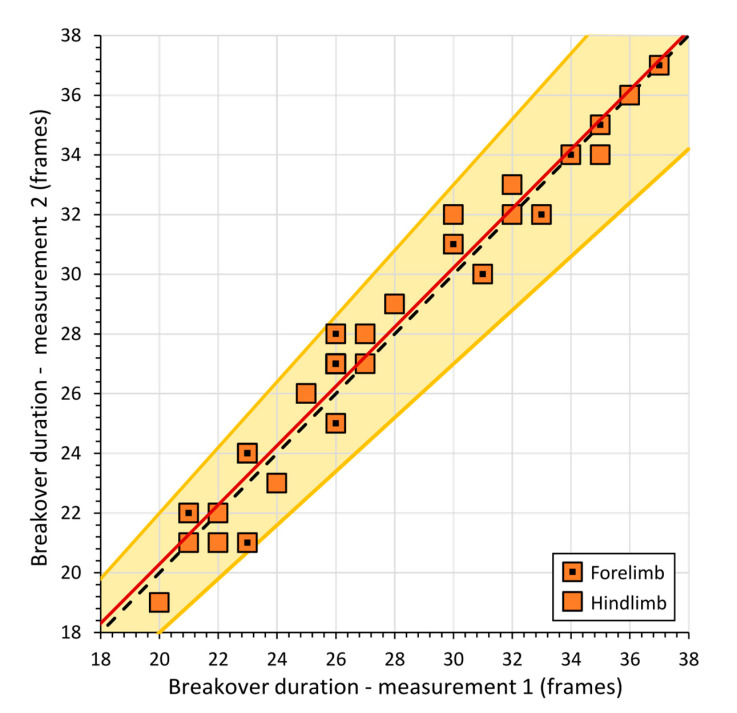
Reproducibility of breakover duration data quantified using Tracker software. Dashed line is a 1:1 line and the shaded band indicates ±10% deviations from this line. The solid red line shows the linear best fit to the combined forelimb and hindlimb data (*y* = 98 *x* + 0.70, *r*^2^ = 0.96, *n* = 30). The mean deviation of the data was ±3%, ±2 S.D. = ±0.05%, which is equivalent to a mean difference in breakover duration of 0.8 ± 1.2 frames (mean ± 2 S.D.).

Duplicate analyses of 30 videos covering 15 forelimb and 15 hindlimb breakover events were selected at random and used to assess the reproducibility and precision of the method. The data from the repeat analyses showed close agreement to a 1:1 relationship (Figure 2). The mean deviation between replicate breakover duration values was 3.0 ± 0.05%, which is equivalent to a mean difference in breakover duration of 0.8 ± 1.2 frames.

To account for an expected influence of gallop speed on breakover duration, the mean gallop speed recorded by the GPS devices between the start and end of the camera set-up was evaluated. Using satellite imagery, the location of the cameras was identified to fall between 52.26579° N, 0.414454° E and 52.26564° N, 0.414711° E on the artificial track, and between 52.2657° N, 0.414237° E and 52.26556° N, 0.414531° E on the turf track. The speed and position of the horse in latitude-longitude space was plotted alongside the camera position to identify the relevant speed data.

### 2.6. Statistics

Mean breakover durations and gallop speeds were first compared between individual limb datasets using two sample *t*-tests, assuming equal variances. Linear regression analysis was also used to assess the relationship between breakover duration and speed using individual run data.

To evaluate the potential impact of shoeing condition or surface type on breakover duration, breakover data were then analysed independently for each limb, in case of any potential differences associated with the asymmetry of the horse’s transverse galloping gait. Mixed models were implemented in SPSS to test for significant differences in breakover duration amongst the different shoe and surface conditions for each limb. Shoe, surface, speed, and ‘shoe*surface’, ‘shoe*speed’ and ‘surface*speed’ interactions were defined as fixed factors. Horse–jockey pair and day were set as random factors. Day was included because weather conditions and hence ground conditions varied across data collection sessions. Speed through cameras was included as a covariate.

The *p* value outputs for the interaction terms of these initial linear mixed models were evaluated. If any *p* values for interaction terms exceeded 0.1, then these terms were removed so ‘final’ models could be run with a reduced number of fixed terms to lower statistical noise. In each case, histograms of models’ residuals were plotted and inspected for normality. The significance threshold in all statistical tests was set at *p* < 0.05.

## 3. Results

**Figure 3:**
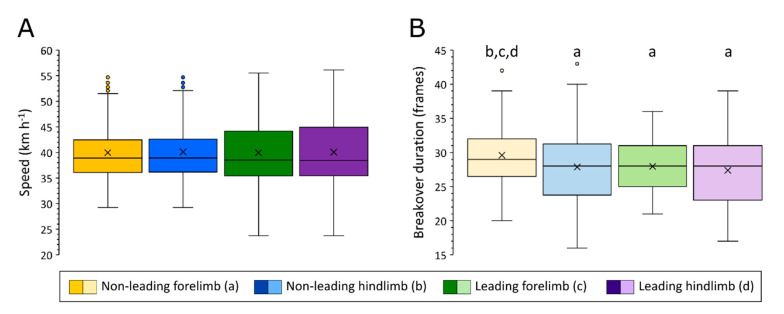
Boxplots illustrating distribution in speed (**A**) and breakover duration (**B**) data across individual limbs. Significant differences (*p* < 0.05) in means (marked ×) between the non-leading forelimb (yellow, ‘a’), the non-leading hindlimb (blue, ‘b’), the leading forelimb (green, ‘c’) and the leading hindlimb (purple, ‘d’) are indicated.

Gallop speeds during runs ranged from 24–56 km h^−1^. Mean gallop speed was consistent across limbs (Figure 3A). However, the gallop speeds at which breakover duration was evaluated were most similar between pairs of leading and non-leading limbs (Figure 3A), reflecting the fact that these limbs were most commonly assessed from the same video footage of gallop runs. Intriguingly, the spread in speed data was not reflected in the spread in breakover data (Figure 3B), with the hindlimbs showing a greater spread (inter-quartile range equal to 7 and 8 frames for the leading and non-leading hindlimbs, respectively) compared to the forelimbs (inter-quartile range equal to 6 and 5 for the leading and non-leading forelimbs, respectively). The greatest difference in mean breakover duration was seen between the non-leading forelimb (30 frames, which is equivalent to 30 ms at 1000 fps), compared to the leading hindlimb (27 frames). Two-tailed t-tests of equal variance indicated significant differences in mean breakover duration were present between the non-leading forelimb and non-leading hindlimb (*p* = 0.014), the two forelimbs (*p* = 0.007), and the non-leading forelimb and leading hindlimb (*p* = 0.002). For all other limb comparisons, *p* ≥ 0.4.

**Figure 4:**
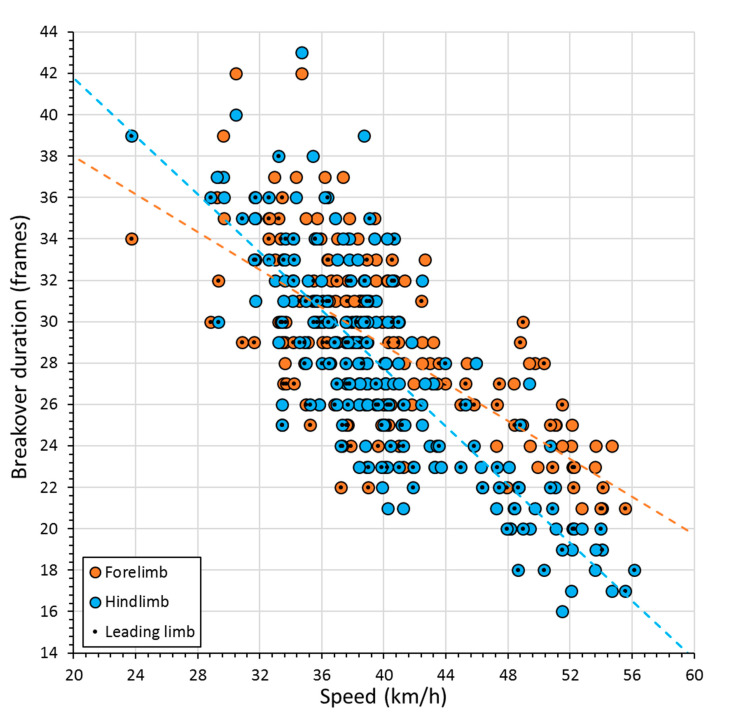
Negative relationship between breakover duration and gallop speed. Data are presented for leading (black dots) and non-leading (open circles) forelimbs (orange) and hindlimbs (blue). The forelimb data fit to a regression line, where y = −0.46 x + 47.12 (r = 0.7). The hindlimb data fit to a regression line, where y = −0.70 x + 55.79 (r = 0.8).

Further investigation into speed and breakover data, based on individual runs, revealed there to be a negative correlation between breakover duration and gallop speed. This relationship was stronger for the hindlimbs (*r*^2^ = −0.67, *p* < 0.05, *n* = 189) than the forelimbs (*r*^2^ = −0.45, *p* < 0.05, *n* = 195) (Figure 4). A separation in forelimb and hindlimb data was also apparent at gallop speeds approximating ≥45 km h^−1^, when a reduction in breakover duration with increasing speed became more marked in the hindlimbs.

The linear mixed models used to assess for an additional influence of either ground surface or horse shoeing condition on breakover durations revealed differences in response amongst the hooves from different limbs. Table 1 summarises the mean breakover duration and speed data sub-divided by shoe–surface combination and limb. The estimated marginal means calculated in the models are presented in Table 2 and Table 3 for surface and shoe, respectively. For the leading forelimb (right forelimb, right lead, *n* = 90), preliminary models indicated that all of the interaction terms had high *p* values (≥0.36). In the final model, including only the main effects (shoe, surface, speed), surface (*p* = 0.024) and speed (*p* < 0.0005) were found to have a significant effect on breakover duration. Mean speed across runs was 40.0 ± 13.4 km h^−1^.

The non-leading forelimb (right fore, left lead, *n* = 105) results were similar to the leading forelimb. In the preliminary model, all interaction terms had high *p* values (≥0.18). The final model (shoe, surface, speed) indicated surface (*p* = 0.002) and speed (*p* < 0.0005) had a significant effect on breakover duration. Mean speed across runs was 40.1 ± 11.9 km h^−1^.

For the leading hindlimb (right hind, right lead, *n* = 87), the preliminary model suggested there was a shoe*surface interaction that might have a significant effect on breakover duration, alongside speed, as *p* values were ≤0.037. However, the final model incorporating shoe, surface, speed and shoe*surface as fixed factors revealed that only surface (*p* = 0.002) and speed (*p* < 0.0005) had a significant effect on breakover duration. Consequently, the data for this hindlimb indicated a similar breakover response to the two forelimbs. Mean speed across runs was 40.1 ± 13.6 km h^−1^.

**Figure 5:**
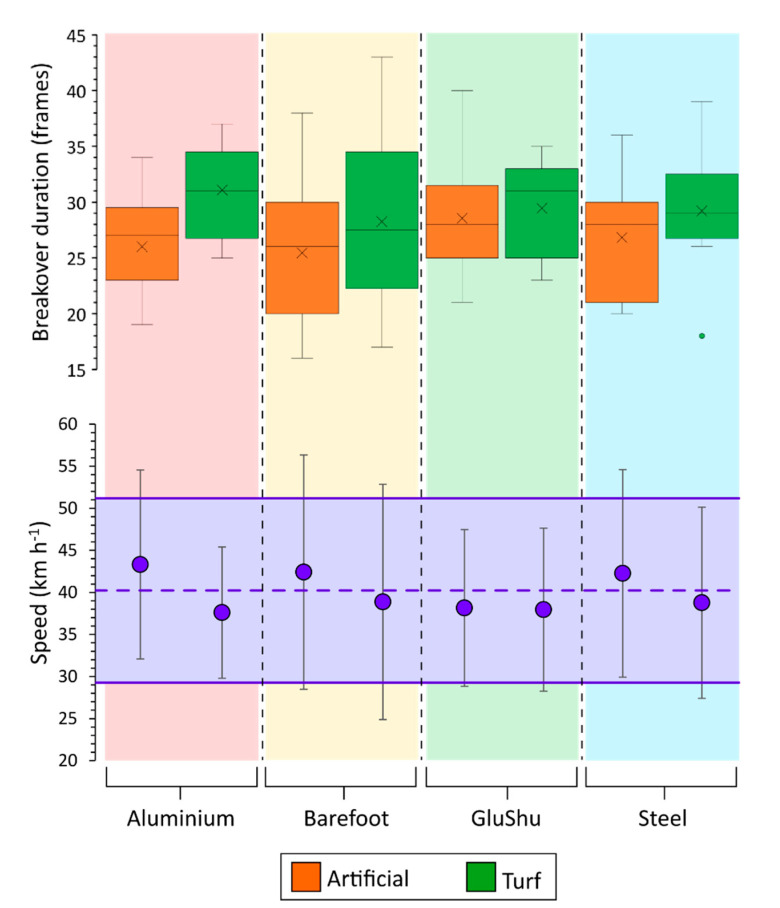
Boxplots illustrating the influence of shoeing condition and surface on breakover duration in the non-leading hindlimb. Data are clustered by shoe type for artificial (orange) and turf (green) data. The mean speed across the data set is illustrated by the dashed purple line, with ±2 S.D. falling within the shaded purple area and bounded by the solid purple lines. The mean speed ±2 S.D. for each shoe–surface category is marked by the purple circles.

For the non-leading hindlimb (right hind, left lead, *n* = 102), the preliminary model suggested shoe, surface and speed may be significant. For the interaction terms, shoe*surface had a high *p* value (0.642) and hence was discounted from the final model, whereas the shoe and surface interactions with speed had *p* values less than 0.1 (0.05 and 0.056, respectively) and hence remained in the final model. The final model (shoe, surface, speed, shoe*speed, surface*speed) indicated that shoe (*p* = 0.025), surface (*p* = 0.04), speed (*p* < 0.0005) and shoe*speed (*p* = 0.023) all had a significant effect on breakover duration, thus setting the results of this hindlimb apart from the other three limbs. Mean speed across runs for this limb was 40.2 ± 11.6 km h^−1^. Boxplots displaying the results of this limb are presented in Figure 5. In all linear mixed models performed, histograms of residuals confirmed normality.

Across limbs, the significant effect of surface type was reflected in an increase in the estimated marginal mean breakover duration on turf compared to the artificial track (Table 2, Figure 5). In diagonal pairs of limbs, the turf-artificial difference was comparable: the non-leading hindlimb and leading forelimb showed a mean difference of 0.7 and 0.8 frames, respectively, whereas for the leading hindlimb and non-leading forelimb, the mean difference was almost double at 1.3 and 1.4 frames, respectively.

A post hoc analysis was performed for the non-leading hindlimb to explore the effect of the four shoeing conditions in more detail at four speed categories of comparable sample size: (1) ‘slow’, 29.24–36.05 km h^−1^ (*n* = 27); (2) ‘medium-1′, 36.17–38.92 km h^−1^ (*n* = 28); (3) ‘medium-2′, 38.93–42.95 km h^−1^ (*n* = 28); and (4) ‘fast’, 43.15–54.68 km h^−1^ (*n* = 27). In this post hoc model, only the eight possible shoe–surface combinations and surface were included as fixed factors. Day and horse–jockey pair were kept as random factors. A Bonferroni correction was applied to pairwise comparisons between shoe–speed categories and significance was set at *p* < 0.05. The speed–shoe categories that were most commonly significantly different to the others in terms of breakover duration were the barefoot condition at ‘slow’ (9/15 comparisons, *p* ≤ 0.047) and ‘fast’ (12/15 comparisons, *p* ≤ 0.008) speed categories. For the ‘fast’ category, the steel, aluminium and GluShu shoes behaved similarly and generally only showed significant differences to the ‘slow’ categories for all shoeing conditions; the aluminium shoe was additionally different to the ‘steel at medium-1 speed’ condition. For the ‘medium-1′ speed category, there were few significant differences amongst the shoeing conditions but the greatest number (3/15) were found for the steel shoe, which was significantly different to ‘fast’ categories for steel, barefoot and aluminium. For the ‘medium-2′ speed category, again there were few significant differences. At ‘slow’ speeds, the steel shoe was only significantly different to the other shoeing conditions when they were compared to ‘fast’ speeds. For the GluShus, in addition to being different to the other shoeing conditions at high speeds they were also different to aluminium and barefoot at ‘medium-2′ speeds. In addition to GluShu, aluminium at ‘slow’ speeds was also significantly different to aluminium and barefoot at the ‘medium-2 category’, as well as all shoeing conditions when ‘fast’. Barefoot in the ‘slow’ category was significantly different to all ‘fast’ and all ‘medium-2′ categories and also to aluminium at the ‘medium-1′ speed. The results of this post hoc analysis are summarised in Table 4.

## 4. Discussion

The findings of this study emphasise that breakover duration in galloping Thoroughbreds can be sensitive to speed, ground surface type and shoeing condition to varying extents depending on the limb being evaluated.

### 4.1. The Role of Speed

The reduction in breakover duration observed with increasing gallop speed is consistent with observations in walk and trot, which highlight breakover duration to be a primarily speed-dependent variable [29]. Our data additionally highlight an interesting distinction between breakover duration in hindlimbs and forelimbs, which is dependent on gallop speed. At low-moderate gallop speeds, breakover duration in forelimbs and hindlimbs appears similar (Figure 4). This may be related to an even distribution of body weight between forelimbs and hindlimbs, as forceplate data indicate that the front:hind loading in the horse approximates 50:50 at a gallop speed of 41 km h^−1^ [30]. For comparison, it is also true that the forelimbs and hindlimbs of dogs belonging to various breeds appear to contribute equally to load support at their low gallop speeds (9.2 ± 0.3 m s^−1^; mean ±1 S.D.) [31]. However, as gallop speeds in the horse increase beyond approximately 45 km h^−1^, our breakover duration data show a greater relative decrease in the hind hooves compared to the fore hooves, i.e., a faster breakover in the hindlimbs (Figure 4). It is interesting to note that hindlimbs have a net accelerative horizontal impulse in galloping dogs [32] and further investigation is needed to understand how a reduced breakover time in the hindlimbs and an increased vertical impulse is beneficial for high gallop speeds in the horse. Perhaps it is efficient for the horse to rotate the toe into the surface faster, so that the forwards push occurs with the accelerative force perpendicular to the solear surface. In addition, it may be related to the need to reduce the likelihood of slippage through increased vertical force when the accelerative force increases.

At high speeds, the front:back body loading distribution has been shown to change in cheetahs and greyhounds [33]. For these animals, it was suggested that being able to support a greater proportion of body weight on the hindlimbs at high speeds might enhance grip for acceleration and manoeuvring using hindlimb musculature [33]. This is also likely to be important for the galloping Thoroughbred, where minimising slipping during propulsive efforts in the hind end may lessen the risk of injury. Acquiring sufficient grip through this biomechanical strategy is likely to be particularly important on high-speed turns. In these instances, friction limits the total horizontal force the horse can apply and governs the balance between forward acceleration and centripetal acceleration [34]. In contrast, the forelimbs may be more involved in leverage and hence the time taken for the hoof to breakover may increase. It would be interesting to investigate this front:hind breakover duration relationship further in racing Thoroughbreds, where top speeds of approximately 19 m s^−1^ (68 km h^−1^) have been reported [35], and hence quite substantially exceed those of the retired Thoroughbreds available for this study. If breakover duration can be used as a proxy for forelimb:hindlimb loading in the horse, our data would predict that the front:hind loading distribution at the horse’s top gallop speed will approximate 34:66 (Figure 4). This would place the horse mid-way between the cheetah (30:70) and the greyhound (38:62) in terms of front:hind body load distribution, when these animals are evaluated at a comparable speed of 18 m s^−1^ [33].

Given the variability between the forelimb and hindlimb data, these results support the notion that breakover in forelimbs and hindlimbs should be evaluated separately [13,29]. However, this study additionally found important differences arose between the individual limbs in response to shoeing condition (see Section 4.3), suggesting a limb-by-limb approach is most appropriate for interpreting data from this asymmetric gait.

### 4.2. The Role of Surface

This study revealed breakover durations were longer on the turf track compared to the artificial surface for all hooves. Ground conditions are commonly the dominant influence on hoof biomechanics [36], and it is possible that the turf versus artificial surface differences masked more subtle effects of shoeing on breakover duration, particularly in the forelimbs and the leading hindlimb.

There are several possible explanations for the surface differences. First, the artificial surface may have offered more grip, as was perceived by the jockeys [25], and thus allowed the horses to push-off more effectively and quickly. Second, artificial surfaces have a high level of elastic deformation [37] and it is likely that this surface returned more energy to the hoof than the turf through elastic rebound, thereby potentially accelerating breakover. Third, it is possible that the softer nature of the artificial track meant the toe rotated into the surface more easily. Preliminary hoof accelerometery data from this group of horses indicate that peak accelerations are higher on the turf compared to the artificial track at impact, which is suggestive of a generally softer artificial surface (Horan et al., unpublished data). Increased loading of the toe at mid-stance on soft surfaces increases forward rotation of the hoof [20], which is also an effect that may hasten breakover. On perhaps firmer turf, the hooves may have been more likely to slip caudally prior to lift-off. Fourth, the turf surface appeared to be more irregular, compared to the frequently harrowed artificial track, and it is plausible that a more uneven surface would result in non-simultaneous impact of the medial and lateral heels. As a result, the hooves would not necessarily rotate around the centre of the toe: an effect that may also act to lengthen the duration of breakover. However, it is a limitation of this study that surface regularity, as well as weather driven chances in surface properties, such as moisture and temperature, e.g., [38,39], were not accounted for and this may have biased results.

Given the influence of surface type on all hooves, it is perhaps unsurprising that peak centre of mass displacements in all directions (cranio-caudal, medio-lateral and dorso-ventral) for the horse were also found to be sensitive to surface type (Horan et al., 2021, in revision). Of particular note, larger vertical displacements of the horses’ centre of mass on the artificial track are consistent with a faster breakover and increased push-off. Our future work seeks to evaluate push-off accelerations from hoof accelerometery data collected concurrently from this study population in detail, to assess whether decreases in breakover duration are matched by increased push-off accelerations, and vice versa. As the majority of racehorses in the UK train on artificial surfaces and race on turf, understanding how and why horse and jockey kinematics differ between these surfaces is relevant for performance and injury prevention.

### 4.3. The Role of Shoeing Condition

Although breakover duration in all limbs was sensitive to speed and surface type, the non-leading hindlimb was additionally found to be sensitive to shoeing condition. There are several possible explanations for this result. As the first limb to strike the ground after the suspension phase of a gallop stride, the non-leading hindlimb needs to do the most accelerative work after the forelimbs have slowed the horse down [40], in addition to overcoming aerodynamic drag [35]. Additionally, the orientation of the horse may be the least constrained after the aerial phase, meaning hindlimbs must stabilise the movement of the horse to enable the forelimbs to strike the ground more predictably. It is also possible that proprioceptive feedback from the non-leading hindlimb influences subsequent activity. The narrower spread in forelimb breakover duration data (Figure 3B) could reflect a more consistent landing orientation in the front hooves compared to the hind hooves. This result is similar to the breakover duration patterns observed in forelimbs of warmblood horses during trotting [41]. However, in the latter study this was attributed to higher loading and an associated reduction in the ability of front hooves to compensate for changes in shoeing or hoof shape. Further investigation into the differing patterns in breakover duration and loading at different gaits, as well as speeds, is therefore warranted (see also Section 4.1). Nevertheless, in the galloping horse, the slightly longer stance period in non-leading hindlimb [42], coupled with increased vertical impulse relative to the leading hindlimb [30], is consistent with the idea of a stabilisation process taking place. Accommodating the additional and potentially unpredictable force imposed by the jockey is also important, and jockey stirrup pressures are known to increase on the opposite side to the leading leg [43]. Hence, the position of the jockey may also drive greater sensitivity of the non-leading hindlimb to external factors, such as shoeing condition.

For the non-leading hindlimb, the greatest number of significant differences occurred between the barefoot hooves and the other three shoeing conditions (26 out of 60 speed–shoe comparisons). However, the majority of these differences were present between the ‘fast’ (12) and ‘slow’ (9) barefoot categories, compared to the other ‘speed–shoe’ categories (Table 4). The barefoot condition is associated with longer breakover durations at slow speeds and more rapid breakover at higher speeds, relative to the other conditions. The BRS horses used here are commonly left barefoot behind and only shod in-front, therefore unfamiliarity to the barefoot condition, as might be expected in a racing Thoroughbred, would be unlikely to explain the results here. At increasing speeds, heel expansion is expected to increase [44], likely reflecting the increase in vertical ground reaction force at mid-stance. In the barefoot condition, it is plausible that a greater percentage of potential energy would be stored, as the heels expand under load to a greater extent, relative to the shod conditions. The subsequent release of this energy may accelerate breakover as gallop speeds increase. In contrast, shoeing can reduce medio-lateral heel movement [45], and therefore is likely to lessen this effect for the three shod conditions.

At lower gallop speeds, when less potential energy is stored in the hoof, perhaps hoof conformation and shoe shape become more important. The more bevelled edge of the shoes, compared to the hoof edge, could act to increase the rate at which the hoof rolls over. Alternatively, given that the aluminium shoe also shows a higher number (7/15) of significant pairwise comparisons at the low speeds, compared to the GluShu (6/15) and steel (4/15) conditions, the mass of the distal limb may be relevant. A reduced distal limb mass in the aluminium and barefoot conditions will lower the energetic cost of locomotion, and may permit increased movement variability. In alignment with this observation, vertical displacement at the horses’ girths also increased in the aluminium and barefoot conditions relative to the steel and GluShu conditions (Horan et al., 2021, in revision). However, it is worth noting the caveat that our breakover dataset includes an uneven number of the different shoeing conditions per speed category in the post hoc analysis; for example, there are less high-speed data for GluShu and the majority of the high-speed data are represented by the aluminium and barefoot conditions on the artificial surface (Supplementary Material Tables S1 and S2).

### 4.4. Individual Variability

The potential effect of individual horse or jockey variation on hoof breakover was accounted for in our statistical modelling by including ‘horse–jockey pair’ as a random factor. It was apparent that several of the horses in this study were used to galloping on one lead more than the other and also showed variability in hoof angles and conformation [25], which could reflect underlying movement asymmetries, perhaps related to anatomy, or biomechanical adaptations. For example, horse–jockey pairs 10 and 14 each completed all trial conditions over a comparable speed range, yet the breakover duration of the former were always longer. Although it is not clear why this difference arose, Horse 10 had a preference to strike off and gallop on the left lead. This horse also had larger hooves, which required larger shoes that would increase distal limb mass. It would be interesting to evaluate the influence of hoof morphology, for example solear surface area, and shoe mass on breakover duration in future work. Although these two horses were of similar height, they also differed in body dimensions and Horse 10 had a body mass approximately 50 kg greater than Horse 14. Based on jockey perception of the horses’ adaptation period in each trial, it also appears that some horses took slightly longer to adapt to some shoeing conditions, particularly barefoot [25]. Further investigation is required to determine how individual horse variation, as differences in conformation or long-term changes to the locomotor system, might bear relevance for breakover times. The latter could arise as the horses adapt to particular environmental working conditions, including track curvature and surface. Such horse variation and its potential effect on hoof kinematics may additionally play out in terms of injury prevalence. For example, racehorses with larger solar surface areas have fewer catastrophic musculoskeletal injuries, including suspensory apparatus failure and cannon bone condylar fracture [46].

In addition, it would be valuable to evaluate how breakover durations might vary between shoeing conditions over the shoeing interval and after training. In this study, all horses’ hooves were trimmed immediately prior to analysis, but hoof shape, including hoof angles and coronary band hoof circumference, has been shown to differ between steel shod and barefoot horses over a seven week shoeing cycle [47]. Periods of race training can also alter hoof shape, such as hoof angles [48] and coronary band hoof circumference [49]. It has been reported that racing Thoroughbreds with upright hooves sustain fewer musculoskeletal injuries and achieve greater success [50]. Reducing the difference between toe and heel angles can also lessen the risk of suspensory apparatus failure [46]. Future studies could therefore seek to understand how the effect of different shoeing cycles and exercise regimens on hoof shape plays out in terms of breakover times.

## 5. Conclusions

This study evaluated whether four shoeing conditions (aluminium, barefoot, GluShu and steel) readily applicable to the galloping Thoroughbred racehorse influenced the duration of hoof breakover on turf and artificial training tracks. Breakover duration was found to be significantly longer on the turf track for both leading and non-leading forelimbs and hindlimbs. In contrast, shoeing condition was only found to affect breakover duration in the non-leading hindlimb, where it also showed a significant interaction with speed. A post hoc evaluation of the shoe–speed interaction across four speed categories for the non-leading hindlimb revealed that shoeing condition had only a limited influence on hoof breakover durations over the central speed range, with pairwise comparisons revealing few significant differences. However, at high and low gallop speeds, the barefoot condition was often significantly faster and slower, respectively, when compared to the other three shoeing conditions. It was hypothesised that greater storage and release of energy from a more expanded barefoot hoof may accelerate breakover at higher gallop speeds, and a less predictable landing orientation for a lighter distal limb may drive more variable and longer breakover times at lower speeds. Breakover duration showed a negative linear relationship with speed, although hindlimb hooves were found to have a greater relative decrease in breakover duration compared to the forelimb hooves at gallop speeds beyond approximately 45 km h^−1^. We proposed that this may be linked to a greater propulsive effort required from the hindlimbs. The front:hind breakover duration ratio followed trends previously measured for front:hind body weight distribution at low-moderate gallop speeds, and extrapolation of the front:hind breakover duration ratio to top racing speeds predicted the transfer of more body weight to the hind end. This prediction would be consistent with the biomechanical strategies previously reported in the galloping cheetah and greyhound. Importantly, the results of this study may have implications for minimising the risk of injury and improving performance in galloping Thoroughbreds.

## Figures and Tables

**Table 1:** Summary of breakover duration and speed data sub-divided by shoe–surface combination and limb. The number of horse–jockey pairs available in the analysis of each condition is stated.

Shoe–Surface Combination	Limb	Number of Observations	Number of Horse–Jockey Pairs	Mean Breakover Duration (Frames) ± 2 S.D.	Mean Speed (km h^−1^) ± 2 S.D.
Aluminium–Artificial	Non-leading forelimb	16	12	27.44 ± 6.41	42.91 ± 11.49
	Non-leading hindlimb	17	12	26.00 ± 8.40	42.95 ± 11.13
	Leading forelimb	14	11	26.79 ± 5.83	43.20 ± 13.02
	Leading hindlimb	14	11	24.86 ± 8.87	43.20 ± 13.02
Aluminium–Turf	Non-leading forelimb	14	7	32.29 ± 8.17	37.59 ± 7.80
	Non-leading hindlimb	14	7	31.07 ± 8.47	37.59 ± 7.80
	Leading forelimb	8	7	29.63 ± 6.41	35.91 ± 7.54
	Leading hindlimb	8	7	31.63 ±6.58	35.91 ± 7.54
Barefoot–Artificial	Non-leading forelimb	18	14	27.17 ± 7.20	42.40 ± 13.93
	Non-leading hindlimb	18	14	25.44 ± 12.10	42.40 ± 13.93
	Leading forelimb	14	12	26.36 ± 7.95	42.76 ± 15.23
	Leading hindlimb	14	12	25.07 ± 12.24	42.65 ± 14.96
Barefoot–Turf	Non-leading forelimb	9	8	31.00 ± 11.92	38.87 ± 13.97
	Non-leading hindlimb	8	8	28.25 ± 16.48	39.61 ± 14.17
	Leading forelimb	10	9	29.10 ± 8.19	37.80 ± 11.52
	Leading hindlimb	10	8	28.70 ± 11.66	39.69 ± 16.31
GluShu–Artificial	Non-leading forelimb	13	10	29.85 ± 10.42	38.15 ± 9.32
	Non-leading hindlimb	13	10	28.54 ± 10.54	38.15 ± 9.32
	Leading forelimb	9	9	28.22 ± 8.47	38.28 ± 10.08
	Leading hindlimb	9	9	27.11 ± 6.36	38.28 ± 10.08
GluShu–Turf	Non-leading forelimb	10	7	32.60 ± 5.75	37.14 ± 8.05
	Non-leading hindlimb	11	7	29.45 ± 8.69	37.94 ± 10.16
	Leading forelimb	12	8	30.25 ± 5.54	35.79 ± 11.15
	Leading hindlimb	11	8	30.45 ± 8.96	35.97 ± 11.62
Steel–Artificial	Non-leading forelimb	12	11	28.58 ± 7.79	42.41 ± 12.74
	Non-leading hindlimb	11	11	26.82 ± 10.07	40.69 ± 11.93
	Leading forelimb	13	11	26.92 ± 8.42	41.71 ± 13.91
	Leading hindlimb	11	11	27.36 ± 10.78	40.87 ± 12.80
Steel–Turf	Non-leading forelimb	13	9	30.15 ± 8.44	38.77 ± 11.36
	Non-leading hindlimb	10	9	29.20 ± 11.11	40.35 ± 10.24
	Leading forelimb	10	7	27.70 ± 9.34	41.25 ± 13.52
	Leading hindlimb	10	7	26.30 ± 10.54	40.92 ± 13.75

**Table 2:** Linear mixed model estimated marginal means for surface effects.

Limb	Surface	Mean	Std. Error	df	95% Confidence Interval (Lower Bound)	95% Confidence Interval (Upper Bound)
Non-leading forelimb	Artificial	28.48	0.73	15.07	26.92	30.04
Non-leading forelimb	Turf	29.83	0.76	17.36	28.23	31.44
Non-leading hindlimb	Artificial	26.90	0.69	16.38	25.44	28.36
Non-leading hindlimb	Turf	27.59	0.73	19.62	26.07	29.11
Leading forelimb	Artificial	27.89	0.79	14.18	26.20	29.59
Leading forelimb	Turf	28.69	0.80	14.81	26.98	30.40
Leading hindlimb	Artificial	27.18	0.75	15.59	25.58	28.77
Leading hindlimb	Turf	28.46	0.76	16.67	26.85	30.08

**Table 3:** Linear mixed model estimated marginal means for shoeing condition effects.

Limb	Shoeing Condition	Mean	Std. Error	df	95% Confidence Interval (Lower Bound)	95% Confidence Interval (Upper Bound)
Non-leading forelimb	Aluminium	29.61	0.78	19.23	27.98	31.24
Non-leading forelimb	Barefoot	29.15	0.78	19.02	27.52	30.77
Non-leading forelimb	GluShu	29.03	0.81	21.94	27.34	30.71
Non-leading forelimb	Steel	28.84	0.79	19.77	27.20	30.49
Non-leading hindlimb	Aluminium	27.23	0.74	21.21	25.69	28.77
Non-leading hindlimb	Barefoot	27.18	0.75	21.85	25.63	28.72
Non-leading hindlimb	GluShu	26.90	0.78	24.75	25.30	28.50
Non-leading hindlimb	Steel	27.67	0.77	23.95	26.09	29.25
Leading forelimb	Aluminium	28.46	0.83	17.14	26.70	30.21
Leading forelimb	Barefoot	28.30	0.81	15.76	26.58	30.03
Leading forelimb	GluShu	28.48	0.83	17.04	26.72	30.23
Leading forelimb	Steel	27.94	0.83	16.90	26.18	29.69
Leading hindlimb	Aluminium	28.15	0.80	19.78	26.47	29.82
Leading hindlimb	Barefoot	27.59	0.78	17.73	25.96	29.22
Leading hindlimb	GluShu	27.90	0.81	20.51	26.22	29.59
Leading hindlimb	Steel	27.64	0.81	20.30	25.96	29.32

**Table 4:** Summary of post hoc results for pairwise comparisons of shoeing conditions at four speed categories.

Shoeing Condition	Speed Category	Number of Significantly Different Shoe–Speed Conditions
Aluminium	Fast	5
Aluminium	Medium-1	2
Aluminium	Medium-2	4
Aluminium	Slow	7
Barefoot	Fast	12
Barefoot	Medium-1	1
Barefoot	Medium-2	4
Barefoot	Slow	9
GluShu	Fast	4
GluShu	Medium-1	1
GluShu	Medium-2	3
GluShu	Slow	6
Steel	Fast	5
Steel	Medium-1	3
Steel	Medium-2	2
Steel	Slow	4

## Data Availability

Data supporting results are presented in the ‘Results’ section of this manuscript.

## Supplementary Materials

The following are available online at https://www.mdpi.com/article/10.3390/ani11092588/s1, Table S1: Raw breakover duration data for the leading limbs, Table S2: Raw breakover duration data for the non-leading limbs.
