# Supplementary material for: Influence of Speed, Ground Surface and Shoeing Condition on Hoof Breakover Duration in Galloping Thoroughbred Racehorses
Source: Animals (Basel). 2021 Sep 3;11(9):2588. doi: 10.3390/ani11092588 (PMC8472780; doi:10.3390/ani11092588)
Supplement: Supplementary file 1 [file animals-11-02588-s001.zip › animals-1359516-SI.pdf]

Supplementary Material

Raw breakover duration data are provided below for leading limbs (Table S1) and non-leading limbs (Table S2).

Table S1. Raw breakover duration data for the leading limbs. Horse ID, Rider ID, Horse–Jockey pair ID and day are consistent with [25]; please note that as data from Horse–Jockey Pair ID-12 were not available for this study, there were 14 horse-jockey pairs involved here. Each row represents data from one gallop run.

Data Collection Date	Shoeing Condition	Surface	Horse ID	Rider ID	Horse–Jockey Pair ID	GPS Speed (km/h)	Forelimb Breakover Duration (frames)	Hindlimb Breakover Duration (frames)
17/01/2019	Barefoot	Artificial	1	1	15	47.43	27	22
17/01/2019	Barefoot	Turf	1	1	15	56.12		18
17/01/2019	Barefoot	Turf	1	1	15	52.17	23	20
17/01/2019	Steel	Artificial	1	1	15	50.83	23	21
17/01/2019	Steel	Turf	1	1	15	54.08	21	19
17/01/2019	Steel	Turf	1	1	15	52.25	23	20
15/02/2019	Barefoot	Turf	2	2	2	40.92	29	30
15/02/2019	Aluminium	Turf	2	2	2	39.10	31	35
15/02/2019	Aluminium	Artificial	2	2	2	45.25	27	26
08/06/2019	Steel	Artificial	3	3	3	48.80	29	25
08/06/2019	Aluminium	Artificial	3	3	3	50.72	25	24
06/07/2019	Barefoot	Artificial	4	3	4	53.95	21	20
06/07/2019	Aluminium	Artificial	4	3	4	55.55	21	17
06/07/2019	Steel	Artificial	4	3	4	54.10	22	
06/07/2019	Barefoot	Artificial	5	3	5	50.70		22
06/07/2019	Barefoot	Artificial	5	3	5	47.90	22	20
03/08/2019	Aluminium	Artificial	6	3	6	48.98	30	20
03/08/2019	Aluminium	Artificial	6	3	6	51.50	26	19
03/08/2019	Barefoot	Artificial	6	3	6	50.32	28	18
03/08/2019	Steel	Artificial	6	3	6	48.42	25	21
03/08/2019	Barefoot	Artificial	7	3	7	52.22	22	
10/08/2019	Aluminium	Artificial	8	4	8	40.43	26	24
10/08/2019	Barefoot	Artificial	8	4	8	36.00	30	28
10/08/2019	Barefoot	Artificial	8	4	8	40.98	24	23
10/08/2019	Steel	Artificial	8	4	8	41.18	25	25
24/08/2019	GluShu	Artificial	9	3	9	46.33	22	22
24/08/2019	Barefoot	Artificial	9	3	9	48.65	22	18
24/08/2019	Steel	Artificial	10	4	10	35.58	34	34
07/09/2019	Steel	Artificial	7	3	7	44.95	26	23
07/09/2019	Aluminium	Artificial	7	3	7	43.95	27	28
07/09/2019	Barefoot	Artificial	11	4	11	33.52	27	33
07/09/2019	Steel	Artificial	11	4	11	34.96	26	31
07/09/2019	Steel	Artificial	11	4	11	37.86	24	32
07/09/2019	Barefoot	Artificial	10	4	10	36.38	33	36
07/09/2019	Aluminium	Artificial	10	4	10	40.65	32	34
28/09/2019	GluShu	Artificial	7	3	7	47.30	26	23
28/09/2019	Barefoot	Artificial	7	3	7	45.80	26	24
28/09/2019	GluShu	Artificial	11	4	11	36.82	26	29
28/09/2019	Aluminium	Artificial	11	4	11	37.68	25	26
28/09/2019	Aluminium	Artificial	11	4	11	34.88	28	29
28/09/2019	Aluminium	Artificial	11	4	11	37.30	24	25
05/10/2019	Barefoot	Turf	8	4	8	36.08	29	31
05/10/2019	GluShu	Turf	8	4	8	33.72	27	

05/10/2019	Aluminium	Turf	8	4	8	36.33	29	31
25/10/2019	Barefoot	Turf	10	4	10	33.23	35	38
25/10/2019	GluShu	Turf	10	4	10	40.50	33	32
11/01/2020	Steel	Turf	4	3	4	41.25	26	24
11/01/2020	Steel	Turf	4	3	4	37.57	25	27
11/01/2020	GluShu	Turf	4	3	4	39.47	26	27
11/01/2020	GluShu	Artificial	4	3	4	34.18	29	32
18/01/2020	Barefoot	Turf	4	3	4	37.62	31	30
18/01/2020	Aluminium	Turf	4	3	4	38.20	30	30
18/01/2020	Barefoot	Turf	13	3	13	33.32	34	30
18/01/2020	Barefoot	Artificial	13	3	13	31.70	33	36
18/01/2020	Aluminium	Artificial	13	3	13	43.53	28	24
18/01/2020	Aluminium	Turf	13	3	13	32.56	35	33
31/01/2020	GluShu	Turf	8	4	8	29.34	32	30
31/01/2020	GluShu	Artificial	8	4	8	37.78	27	27
31/01/2020	GluShu	Turf	11	4	11	33.68	30	34
31/01/2020	GluShu	Turf	11	4	11	23.74	34	39
31/01/2020	GluShu	Turf	11	4	11	30.85	29	35
31/01/2020	Barefoot	Turf	11	4	11	37.55	29	28
31/01/2020	Barefoot	Turf	11	4	11	31.62	29	33
31/01/2020	Aluminium	Turf	11	4	11	34.23	27	33
31/01/2020	Aluminium	Turf	11	4	11	28.84	30	36
31/01/2020	Steel	Turf	11	4	11	35.70	30	31
07/02/2020	Aluminium	Artificial	1	3	1	34.57	31	29
07/02/2020	Aluminium	Turf	1	3	1	38.42	31	29
07/02/2020	Barefoot	Turf	1	3	1	38.22	30	29
07/02/2020	Barefoot	Artificial	1	3	1	40.28	29	26
07/02/2020	Steel	Artificial	3	3	3	32.57	34	36
07/02/2020	Steel	Turf	3	3	3	38.75	32	32
08/02/2020	Steel	Artificial	13	3	13	35.45	32	30
08/02/2020	Steel	Turf	13	3	13	40.50	30	
08/02/2020	Steel	Turf	13	3	13	34.14	34	34
08/02/2020	GluShu	Turf	13	3	13	37.70	32	29
08/02/2020	GluShu	Artificial	13	3	13	36.47	31	28
08/02/2020	Barefoot	Artificial	14	3	14	33.45	25	25
08/02/2020	Barefoot	Turf	14	3	14	37.23	22	24
08/02/2020	Steel	Turf	14	3	14	41.88	23	22
08/02/2020	Steel	Artificial	14	3	14	39.00	22	23
14/02/2020	GluShu	Artificial	14	3	14	35.22	25	26
14/02/2020	GluShu	Turf	14	3	14	40.20	26	23
14/02/2020	Aluminium	Turf	14	3	14	39.60	24	26
14/02/2020	Aluminium	Artificial	14	3	14	39.85	25	23
14/03/2020	GluShu	Artificial	1	3	1	36.96	32	27
14/03/2020	GluShu	Turf	1	3	1	42.43	31	26
14/03/2020	Steel	Turf	1	3	1	36.40	33	30
14/03/2020	Steel	Artificial	1	3	1	38.57	28	
14/03/2020	GluShu	Artificial	6	3	6	33.42	36	30
14/03/2020	GluShu	Turf	6	3	6	38.90	30	29
14/03/2020	GluShu	Turf	6	3	6	38.90	33	31

Table S2. Raw breakover duration data for the non-leading limbs. Horse ID, Rider ID and Horse–Jockey pair ID and day are consistent with [25]; please note that as data from Horse–Jockey Pair ID-12 were not available for this study, there were 14 horse–jockey pairs involved here. Each row represents data from one gallop run.

Day	Shoeing Condition	Surface	Horse ID	Rider ID	Horse–Jockey Pair ID	GPS Speed (km/h)	Forelimb Breakover Duration (frames)	Hindlimb Breakover Duration (frames)
17/01/2019	Barefoot	Artificial	1	1	15	49.40	24	20
17/01/2019	Barefoot	Turf	1	1	15	54.68	24	17
17/01/2019	Steel	Artificial	1	1	15	51.08	25	20
17/01/2019	Steel	Turf	1	1	15	53.59	23	18
15/02/2019	Barefoot	Artificial	2	2	2	41.80	26	29
15/02/2019	Barefoot	Artificial	2	2	2	37.94	29	30
15/02/2019	Barefoot	Artificial	2	2	2	33.00	33	32
15/02/2019	Barefoot	Turf	2	2	2	40.05	28	27
15/02/2019	Aluminium	Turf	2	2	2	39.50	32	30
15/02/2019	Aluminium	Turf	2	2	2	29.64	39	37
15/02/2019	Aluminium	Artificial	2	2	2	43.15	27	27
08/06/2019	Barefoot	Artificial	3	3	3	35.90	34	30
08/06/2019	Barefoot	Artificial	3	3	3	48.38	27	21
08/06/2019	Barefoot	Artificial	3	3	3	53.65	24	19
08/06/2019	Steel	Artificial	3	3	3	49.75	28	21
08/06/2019	Aluminium	Artificial	3	3	3	51.03	25	22
08/06/2019	Aluminium	Artificial	3	3	3	49.37	28	27
08/06/2019	Aluminium	Artificial	3	3	3	45.92	28	28
06/07/2019	Barefoot	Artificial	4	3	4	52.07	24	17
06/07/2019	Aluminium	Artificial	4	3	4	52.75	21	20
06/07/2019	Steel	Artificial	4	3	4	49.90	23	
06/07/2019	Barefoot	Artificial	5	3	5	48.17	20	20
06/07/2019	Aluminium	Artificial	5	3	5	43.28	23	23
06/07/2019	Aluminium	Artificial	5	3	5	43.68		23
03/08/2019	Aluminium	Artificial	6	3	6	52.10	25	19
03/08/2019	Barefoot	Artificial	6	3	6	51.50	24	16
03/08/2019	Steel	Artificial	6	3	6	46.27		23
03/08/2019	Steel	Artificial	6	3	6	48.90	25	
10/08/2019	Aluminium	Artificial	8	4	8	38.83	26	24
10/08/2019	Barefoot	Artificial	8	4	8	40.30	25	26
10/08/2019	Steel	Artificial	8	4	8	40.10	27	27
24/08/2019	GluShu	Artificial	9	3	9	38.40	27	26
24/08/2019	GluShu	Artificial	9	3	9	48.70	22	22
24/08/2019	Barefoot	Artificial	9	3	9	47.25	24	21
24/08/2019	GluShu	Artificial	10	4	10	37.75	32	33
24/08/2019	GluShu	Artificial	10	4	10	36.88	31	35
24/08/2019	GluShu	Artificial	10	4	10	30.44	42	40
24/08/2019	Steel	Artificial	10	4	10	34.32	37	36
07/09/2019	Steel	Artificial	7	3	7	45.35	28	
07/09/2019	Aluminium	Artificial	7	3	7	42.95	28	24
07/09/2019	Barefoot	Artificial	11	4	11	33.62	28	33
07/09/2019	Steel	Artificial	11	4	11	36.56		28
07/09/2019	Barefoot	Artificial	10	4	10	35.42	31	38
07/09/2019	Aluminium	Artificial	10	4	10	39.50	33	31
07/09/2019	Aluminium	Artificial	10	4	10	40.20	32	34
28/09/2019	GluShu	Artificial	7	3	7	43.40	27	24
28/09/2019	Barefoot	Artificial	7	3	7	42.45	29	25
28/09/2019	GluShu	Artificial	11	4	11	35.84	26	26
28/09/2019	Aluminium	Artificial	11	4	11	38.45	28	27
05/10/2019	Steel	Turf	8	4	8	38.88	23	27

05/10/2019	Steel	Turf	8	4	8	35.13	29	32
05/10/2019	Barefoot	Turf	8	4	8	38.93	27	30
05/10/2019	GluShu	Turf	8	4	8	33.64	29	32
05/10/2019	Aluminium	Turf	8	4	8	35.58	31	30
25/10/2019	Steel	Turf	10	4	10	38.52	31	
25/10/2019	Steel	Turf	10	4	10	38.73	32	39
25/10/2019	Steel	Turf	10	4	10	37.77	35	34
25/10/2019	Steel	Turf	10	4	10	29.34	37	
25/10/2019	Barefoot	Turf	10	4	10	34.70	42	43
25/10/2019	Barefoot	Turf	10	4	10	32.96	37	
25/10/2019	GluShu	Turf	10	4	10	37.00	34	33
25/10/2019	GluShu	Turf	10	4	10	35.70	35	34
25/10/2019	Aluminium	Turf	10	4	10	36.17	37	36
25/10/2019	Aluminium	Turf	10	4	10	39.40	35	34
25/10/2019	Aluminium	Turf	10	4	10	38.30	34	33
25/10/2019	Aluminium	Turf	10	4	10	37.38	37	34
25/10/2019	Aluminium	Turf	10	4	10	42.47	28	32
11/01/2020	Steel	Turf	4	3	4	38.92	29	31
11/01/2020	GluShu	Turf	4	3	4	48.05		23
11/01/2020	GluShu	Turf	4	3	4	31.66	36	35
11/01/2020	GluShu	Artificial	4	3	4	36.57	32	30
18/01/2020	Barefoot	Turf	4	3	4	38.88	31	28
18/01/2020	Aluminium	Turf	4	3	4	38.44	31	27
18/01/2020	Barefoot	Turf	13	3	13	29.68	35	36
18/01/2020	Barefoot	Artificial	13	3	13	38.38	29	28
18/01/2020	Aluminium	Artificial	13	3	13	34.12	32	31
18/01/2020	Aluminium	Artificial	13	3	13	38.50	28	30
18/01/2020	Aluminium	Turf	13	3	13	41.28	28	26
18/01/2020	Aluminium	Turf	13	3	13	40.02	28	28
18/01/2020	Aluminium	Turf	13	3	13	29.24	36	37
31/01/2020	GluShu	Turf	8	4	8	35.98		32
31/01/2020	GluShu	Artificial	8	4	8	33.20	30	29
31/01/2020	Steel	Turf	11	4	11	32.64	35	
07/02/2020	Aluminium	Artificial	1	3	1	37.98	29	29
07/02/2020	Aluminium	Turf	1	3	1	38.93	30	26
07/02/2020	Barefoot	Turf	1	3	1	39.90	30	22
07/02/2020	Barefoot	Artificial	1	3	1	40.63	29	27
07/02/2020	Steel	Artificial	3	3	3	36.05	30	30
07/02/2020	Steel	Turf	3	3	3	40.12	28	28
08/02/2020	Steel	Artificial	13	3	13	33.52	33	31
08/02/2020	Steel	Turf	13	3	13	43.20	29	27
08/02/2020	GluShu	Turf	13	3	13	37.70	34	29
08/02/2020	GluShu	Turf	13	3	13	31.70	35	31
08/02/2020	GluShu	Artificial	13	3	13	37.56	31	30
08/02/2020	Barefoot	Artificial	14	3	14	33.43	29	26
08/02/2020	Barefoot	Turf	14	3	14	40.07	25	23
08/02/2020	Steel	Turf	14	3	14	36.96	29	26
08/02/2020	Steel	Artificial	14	3	14	40.25	26	21
14/02/2020	GluShu	Artificial	14	3	14	41.25	23	21
14/02/2020	GluShu	Turf	14	3	14	41.94	27	23
14/02/2020	Aluminium	Turf	14	3	14	39.97	26	25
14/02/2020	Aluminium	Artificial	14	3	14	38.42	26	23
14/03/2020	GluShu	Artificial	1	3	1	40.98	30	27
14/03/2020	GluShu	Turf	1	3	1	42.63	33	27
14/03/2020	Steel	Turf	1	3	1	40.25	32	30
14/03/2020	Steel	Artificial	1	3	1	40.93	30	28
14/03/2020	Steel	Artificial	1	3	1	38.72	31	30
14/03/2020	GluShu	Artificial	6	3	6	34.96	35	28

14/03/2020	GluShu	Turf	6	3	6	41.32	32	25
14/03/2020	GluShu	Turf	6	3	6	38.10	31	
